# Baseline Anti-Dengue IgG Seroprevalence in a Pediatric Brazilian Population Before TAK-003 Vaccination

**DOI:** 10.4269/ajtmh.25-0355

**Published:** 2025-11-25

**Authors:** Amanda Maria Miguel Bortuluzi, Paulo César Pereira dos Santos, Luana Clemm Kuhnen Anschau, Andrea da Silva Santos, Daniel Tsuha, Devanildo de Souza Santos, William M. de Souza, Roberto Dias de Oliveira, Julio Croda

**Affiliations:** ^1^ Post Graduate Program in Health Sciences, Federal University of Grande Dourados, Dourados, Brazil;; ^2^ Post Graduate Program in Infectious and Parasitic Diseases, Federal University of Mato Grosso do Sul, Campo Grande, Brazil;; ^3^ Health Sciences Faculty, Federal University of Grande Dourados, Dourados, Brazil;; ^4^ Oswaldo Cruz Foundation-Mato Grosso do Sul, Campo Grande, Brazil;; ^5^ Municipal Health Department of Dourados, Dourados, Brazil;; ^6^ Department of Microbiology, Immunology, and Molecular Genetics, College of Medicine, University of University of Kentucky, Lexington, Kentucky;; ^7^ Nursing Course, State University of Mato Grosso do Sul, Dourados, Brazil

## Abstract

Dengue remains a major public health challenge in Brazil, with recurrent outbreaks placing a strain on healthcare systems. The TAK-003 vaccine offers a promising control strategy, particularly for children. This cross-sectional study evaluated the seroprevalence of anti-dengue IgG in 643 children and adolescents aged 4–16 years in Dourados, Brazil, before being administered the TAK-003 vaccine. Capillary blood samples were collected on filter paper and analyzed using ELISA; sociodemographic data were obtained through questionnaires. Overall, 20.5% of participants were seropositive. Seropositivity increased with age, with 27.7% among those aged 13–16 years testing positive compared with 13.4% among children aged 4–8 years. Male sex was associated with lower IgG levels. The age-related increase in anti-dengue IgG seroprevalence reflects cumulative exposure to the virus during childhood and adolescence, highlighting the need for immunization strategies tailored to age. These findings emphasize the importance of baseline anti-dengue IgG seroprevalence data to guide vaccination, assess effectiveness, and monitor antibody-dependent enhancement risks.

## INTRODUCTION

Dengue virus (DENV) is a mosquito-borne pathogen transmitted to humans mainly by *Aedes aegypti* mosquitoes. DENV infection can cause dengue fever, a major public health challenge responsible for 96 million cases and 40,000 deaths annually. Over 3.9 billion people in 120 countries live at risk of DENV infection.^1^ Brazil has long been hyperendemic for all DENV serotypes (DENV-1 to DENV-4), with over 6.5 million confirmed cases and over 5,600 deaths reported in 2024.^1^ TAK-003, a live attenuated tetravalent dengue vaccine, is effective against all four dengue serotypes and does not require prior serological screening, making it suitable for large-scale use.^2^^,^^3^ In 2024, Dourados City, located in Mato Grosso do Sul State in Brazil, became the first city in the world to implement a mass vaccination campaign with TAK-003 for individuals aged 4 to 60 years.^4^ Importantly, seroprevalence studies are essential to assess prior anti-dengue IgG exposure and guide vaccination strategies in endemic areas.^5^^,^^6^ In this study, we evaluated anti-dengue IgG seroprevalence in children and adolescents aged 4 to 16 years in Dourados before the TAK-003 vaccination, providing insights into age susceptibility and guiding public health interventions.

## MATERIALS AND METHODS

This cross-sectional study was conducted in Dourados, Mato Grosso do Sul, Brazil (22°13′18.54″S, 54°48′23.09″W), a municipality with 260,640 inhabitants.^7^ Participants included children and adolescents aged 4 to 16 years attending 31 Basic Health Units (BHU) to receive their first dose of the TAK-003 vaccine between February and June 2024. Inclusion criteria were the following: residence in Dourados, age between 4 and 16 years, availability to have a capillary blood sample collected before the TAK-003 vaccination, and eligibility for blood collection. Exclusion criteria included immunosuppression, pregnancy, or incomplete data.

After informed consent was obtained from legal guardians, sociodemographic data were collected via questionnaires. Capillary blood was collected on Whatman 903 filter paper cards via a finger prick, dried for 12–24 hours, and stored at 2–8°C. A 6-mm disk per sample was eluted in 500 *µ*L phosphate-buffered saline with 0.5% Tween 20 at 37°C for 1 hour. Anti-dengue IgG was detected using the ELISA classic Dengue Virus IgG kit (SERION Diagnostics, Wurzburg, Germany), with absorbance measured at 405 nm. The SERION ELISA Dengue Virus IgG test was performed according to the manufacturer’s instructions, including sample preparation, testing procedures, and result interpretation. Samples were classified as positive, negative, or borderline based on optical density values relative to the mean optical density of the standard serum, as specified in the quality control certificate for each kit lot. Borderline results were included in the analysis. Seropositivity was calculated as the number of positive samples divided by the total number of participants tested, including borderline cases.

Data were managed in the Research Electronic Data Capture database software (REDCap. Nashville, TN). Nonparametric tests (Kruskal-Wallis, Mann-Whitney) were used to evaluate associations between anti-dengue IgG levels and the variables age and sex. Quantile regression was used to model variation in IgG levels, with age and sex included as covariates. Proportions and 95% confidence intervals were calculated using the Wilson score method without continuity correction. Sample size (*n* = 600) was calculated assuming a 50% anti-dengue IgG seroprevalence with a 5% error margin. The study was approved by the State University of Mato Grosso do Sul Ethics Committee (protocol: 6.616.686, January 18, 2024) in accordance with the Declaration of Helsinki.

## RESULTS

Among the 643 participants (mean age 10 years, SD 3.4), 50.2% were female and 49.8% were male. The age distribution was relatively balanced, with 36.1% aged 4–8 years, 36.4% aged 9–12 years, and 27.5% aged 13–16 years. Regarding race, 67.7% identified as White, 29.4% as mixed race (pardo), and the remainder as Black (1.3%), Asian (0.6%), or Indigenous (0.6%). The majority (93.9%) reported no known history of dengue infection, highlighting the limitations of self-reported data in assessing prior exposure. A total of 3.2% had reported a previously confirmed dengue episode, and 2.7% reported suspected cases later ruled out (Table 1).

**Table 1:** Sociodemographic characteristics of the participants

Variable	*n* (%)	95% CI
Age group		
4–8 years	232 (36.1)	32.4–39.8
9–12 years	234 (36.4)	32.7–40.1
13–16 years	177 (27.5)	24.1–31.0
Sex		
Female	323 (50.2)	46.4–54.1
Male	320 (49.8)	45.9–53.6
Race/color*		
White	435 (68.1)	64.5–71.7
Mixed	188 (29.4)	25.9–33.0
Black	8 (1.3)	0.4–2.1
Asian	4 (0.6)	0.0–1.2
Indigenous	4 (0.6)	0.0–1.2
Previous infection with dengue		
Unknown	604 (93.9)	91.8–95.5
Confirmed	21 (3.2)	2.1–4.9
Discarded	18 (2.7)	1.7–4.3

CI = confidence interval; *n* (%) refers to the number and percentage of participants.

Total participants = 643.

* Race/color data were unavailable for 4 participants; hence, total *N* = 639 for this variable.

**Figure 1.**
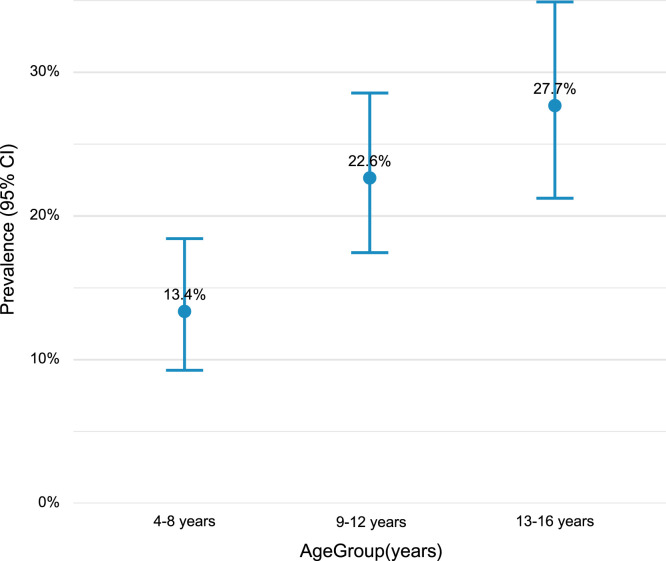
Prevalence of positive IgG by age group.

Overall, 20.5% of participants tested positive for anti-dengue IgG (132/643; 95% CI: 17.4–23.7). Seropositivity increased with age: 13.4% (27/201; 95% CI: 9.5–18.3) in the 4–8 age group, 22.6% (52/230; 95% CI: 16.2–26.5) in the 9–12 age group, and 27.7% (59/213; 95% CI: 21.6–33.1) in the 13–16 age group (Figure 1). Among the 643 participants, 34.1% (219/643) had borderline results. For the Brazilian priority vaccination age group (10–14 years), anti-dengue IgG seroprevalence antibodies were 28.4% (50/176; 95% CI: 22.26–35.48). Kruskal-Wallis testing revealed significant differences in IgG levels across age groups (χ^2^ = 11.7, *P* = 0.003), with the 13–16 age group having significantly higher IgG levels compared with the 4–8 group (Mann-Whitney, *P* = 0.004).

**Figure 2.**
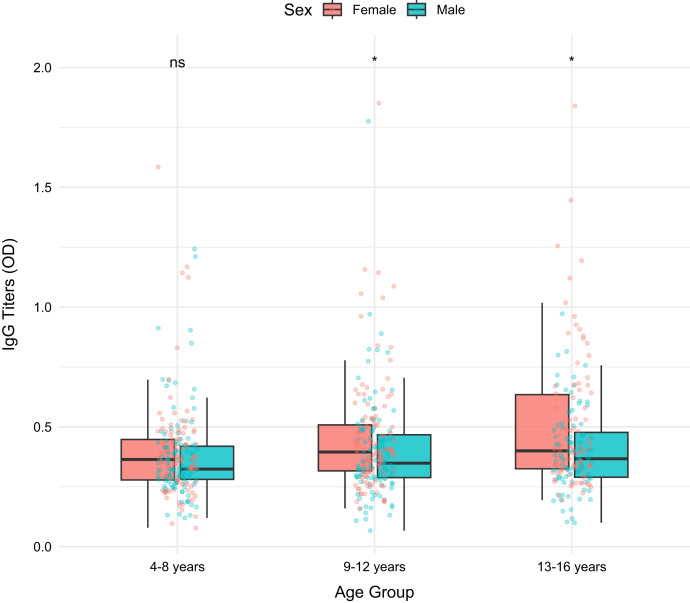
Association of sex and age group with median IgG levels.

Median IgG levels, expressed as units per milliliter (U/mL), were significantly higher in females (0.383, interquartile range [IQR]: 0.305–0.492) than in males (0.344, IQR: 0.280–0.462; Wilcoxon test, *P* = 0.002). Quantile regression analysis confirmed these findings: males had 0.040 U/mL lower IgG levels (95% CI: −0.059 to −0.012), and adolescents aged 13–16 had 0.039 U/mL higher IgG levels (95% CI: 0.012–0.067) compared with children aged 4–8 years, after adjusting for age and sex as covariates (Figure 2).

## DISCUSSION

This study demonstrates that the progressive increase in anti-dengue IgG antibody seroprevalence with age reflects cumulative exposure to DENV in hyperendemic areas. In Mexico, dengue seroprevalence among children aged 0–5 years was 26.9%, increasing to 43.9% in those aged 6–8 years, and reaching 61.4% among children aged 9–15 years, indicating a greater cumulative risk of DENV exposure with increasing age.^8^ A similar age-related pattern was observed in Medellín, Colombia, where seroprevalence reached 61% among children aged 9 to 12 and exceeded 70% only among individuals older than 21 years.^9^ In Brazil, DENV has been circulating for decades, and age-stratified seroprevalence data from different regions suggest that coverage among adolescents aged 10–14 years may still fall below the 70% threshold recommended for dengue vaccine introduction.^10^ This highlights the importance of administering the two doses of the TAK-003 vaccine regimen to protect individuals at risk of primary infection.^11^

Our study also demonstrated that sex differences may influence anti-dengue IgG seroprevalence levels, with females showing higher antibody concentrations than males. However, the underlying reason behind higher IgG levels in women remains undetermined. These elevated IgG levels may result from a stronger immune response, as observed in previous studies.^12^^–^^15^ Some studies suggest that factors such as time spent outdoors or household exposure to *Aedes* mosquitoes may contribute to infection risk, regardless of sex.^16^ Moreover, females often exhibit higher IgG concentrations after being vaccinated for measles, mumps, and rubella, likely due to hormonal influences.^12^^–^^15^ Although dengue seroprevalence does not differ significantly between sexes, other factors, such as having family members with prior dengue infection, may reflect higher household exposure to *Aedes* mosquitoes, thereby increasing the likelihood of infection and, consequently, seropositivity.^16^

These findings indicate it is essential to consider the differences in vaccine efficacy between seropositive and seronegative individuals when designing immunization programs. The TAK-003 vaccine, administered in a two-dose regimen, showed an overall efficacy of 61.2% against virologically confirmed dengue and 84.1% against hospitalized cases over 4.5 years of follow-up. Efficacy was higher among seropositive individuals (ranging from 66.3% to 80.4%, depending on the serotype), but was notably lower in seronegative individuals (53.5%), with probably no efficacy against DENV-3 serotype and unknown protection against DENV-4, possibly because of the small number of cases.^2^^,^^17^ The potential risk of aggravated disease due to serotypes 3 and 4 in seronegative vaccinated children cannot be ruled out.^2^^,^^17^

Furthermore, the potential risk of antibody-dependent enhancement, especially among seronegative individuals, remains a theoretical concern in dengue vaccination.^11^^,^^17^ Although TAK-003 trials show no clear safety signals, ongoing monitoring and seroprevalence data are vital to ensure safe immunization in endemic areas.^1^

## CONCLUSION

The age-related progression of anti-dengue IgG seroprevalence highlights the cumulative exposure to the DENV during childhood and adolescence and reflects the dynamics of dengue transmission in pediatric populations. These findings reinforce the importance of immunization strategies that account for age-specific susceptibility to ensure broader vaccination coverage and more effective interventions. These findings underscore the critical importance of generating robust anti-dengue IgG seroprevalence data before vaccine introduction. Such data are essential to create age-specific immunization strategies and provide context for future assessments of vaccine effectiveness in real-world settings. Moreover, longitudinal monitoring of vaccine performance should consider the potential risk of antibody-dependent enhancement, particularly in relation to the serotype-specific dynamics of dengue circulation. Thus, these findings provide a valuable regional contribution confirming the age- and sex-specific trends in anti-dengue IgG levels, as observed in other Latin American studies, and reinforce the importance of baseline data for evaluating vaccine impact.
